# Under the volcano: phylogeography and evolution of the cave-dwelling *Palmorchestia hypogaea *(Amphipoda, Crustacea) at La Palma (Canary Islands)

**DOI:** 10.1186/1741-7007-6-7

**Published:** 2008-01-31

**Authors:** Carlos Villacorta, Damià Jaume, Pedro Oromí, Carlos Juan

**Affiliations:** 1 Departamento Biologia, Universitat de les Illes Balears, 07122 Palma de Mallorca, Spain; 2 IMEDEA (CSIC-UIB) Instituto Mediterráneo de Estudios Avanzados, 07190 Esporles, Mallorca, Spain; 3 Departamento Biología Animal, Universidad de La Laguna, 38205 La Laguna, Tenerife, Spain

## Abstract

**Background:**

The amphipod crustacean *Palmorchestia hypogaea *occurs only in La Palma (Canary Islands) and is one of the few terrestrial amphipods in the world that have adapted to a strictly troglobitic life in volcanic cave habitats. A surface-dwelling closely related species (*Palmorchestia epigaea*) lives in the humid laurel forest on the same island. Previous studies have suggested that an ancestral littoral *Orchestia *species colonized the humid forests of La Palma and that subsequent drought episodes in the Canaries reduced the distribution of *P. epigaea *favouring the colonization of lava tubes through an adaptive shift. This was followed by dispersal via the hypogean crevicular system.

**Results:**

*P. hypogaea *and *P. epigaea *did not form reciprocally monophyletic mitochondrial DNA clades. They showed geographically highly structured and genetically divergent populations with current gene flow limited to geographically close surface locations. Coalescence times using Bayesian estimations assuming a non-correlated relaxed clock with a normal prior distribution of the age of La Palma, together with the lack of association of habitat type with ancestral and recent haplotypes, suggest that their adaptation to cave life is relatively ancient.

**Conclusion:**

The data gathered here provide evidence for multiple invasions of the volcanic cave systems that have acted as refuges. A re-evaluation of the taxonomic status of the extant species of *Palmorchestia *is needed, as the division of the two species by habitat and ecology is unnatural. The information obtained here, and that from previous studies on hypogean fauna, shows the importance of factors such as the uncoupling of morphological and genetic evolution, the role of climatic change and regressive evolution as key processes in leading to subterranean biodiversity.

## Background

Subterranean ecosystems are considered natural laboratories to study the effect of temporal and spatial isolation on genetic divergence [1]. Environmental stability, permanent darkness and oligotrophy are characteristics of caves, either terrestrial (inhabited by so-called troglobites) or sub-aquatic (occupied by stygobionts). The subterranean environment puts their inhabitants under stress conditions, including "perpetual darkness and humidity, lack of some environmental cues, complex mazelike living space, stressful gas mixtures, patchy food resources, barren rocky substrates and occasional flooding" [2].

The evolutionary patterns of the hypogean fauna are barely known, and precise data on the genetic and biogeographic processes associated with the adaptation to this habitat have only recently begun to be gathered [3-13]. Adaptation to hypogean conditions involves a series of physiological, morphological and behavioural changes collectively known as troglomorphy [14,15]. These adaptations are common to animals as diverse as arthropods, flatworms and vertebrates, suggesting that they are the result of evolutionary convergence caused by living under similar selective pressures to those posed by the hypogean habitat [1,16]. Some of the most studied characteristics in that respect are eye reduction and the loss of pigmentation (body and eye pigments) that Darwin [17] attributed to regressions caused by disuse. Nevertheless, adaptation to these conditions seems to involve also other 'non-regressive' morphological changes, such as elongation of the body, antennae and legs of insects or crustaceans and the exacerbation of sensory organs (e.g. lateral receptors in fish or copepod antennules). Extreme convergence in cave life can also obscure species relationships and their geographical limits [3].

Gammaridean amphipod crustaceans have a considerable number of subterranean stygobiont species in many regions of the world [18]. The global subterranean amphipod fauna comprises more than 700 species embracing 36 families and 138 genera [18,19]. However, amphipods of the family Talitridae (sandhoppers and landhoppers) are the only representatives of the order with strictly terrestrial species, being mainly soil inhabitants of beaches and tropical humid forests. Of these, only two species are known to have adapted to a strictly troglobitic life in volcanic cave habitats: *Spelaeorchestia koloana *[20] in Kaua'i (Hawaiian Islands) and *Palmorchestia hypogaea *[21] in the Canary Islands. A third species, *Orchestia remyi *(accepted as *O. roffoensis *Wildish, 1969) from Corsica, is microphthalmous and presumably has adapted to subterranean life in karstic caves. *P. hypogaea *is exclusively found in lava tubes on the island of La Palma showing troglobitic morphological characters and lifestyle. These include strong eye reduction (significantly fewer partly depigmented and smaller ommatidia than other landhoppers), the complete loss of body pigmentation and very elongated appendages [21,22]. *P. hypogaea *is also one of the few hypogean taxa for which an epigean occulated, pigmented close relative species (*P. epigaea*) is known to occur nearby, in the litter of the humid laurel forest on the same island [21].

Stygobiont amphipods display biogeographic patterns caused by past episodes of colonization by epigean fresh or marine/brackish water ancestors. Adaptive shifts, stream capture, regression of marine embayments and fluctuating sea levels have been recognized as the main driving forces underlying these biogeographic patterns [19]. In landhoppers, Stock [21] suggested an evolutionary scenario for the adaptation of *P. epigaea *to the lava tubes of La Palma. Given the similarity of some morphological characters of the forest-dwelling *P. epigaea *with marine/supralittoral extant *Orchestia *species such as *O. gammarellus*, he proposed that an ancestral littoral *Orchestia *species once colonized the humid forests of La Palma. The recent advent of relative drought episodes in the Canaries would have reduced the previously wider distribution of *P. epigaea *to dark, humid forest pockets remaining at altitudes between 500 and 700 m, especially in the more humid northern and north-eastern face of the island. Drought episodes have been deduced from analyses of Fuerteventura (Canary Islands) paleodunes [23], showing a first cycle at 1.7–1.8 million years ago (MYA) and more recent ones dated from 75,000 to 90,000 years ago, and around 15,000 and 3,640 years before present. These episodes could have affected the island of La Palma, thus creating selection pressures favouring the colonization of deep crevices and lava tubes with enough humid conditions, through an adaptive shift. Alternatively, local extinction of epigean ancestors by the drought episodes mentioned above, and survival of populations in the hypogean habitat and dark humid areas could explain the observed pattern (consistent with the climate relict hypothesis proposed in [24]). In any case, two evolutionary transitions, one from the littoral to inland environment, followed by another from forest to cave habitats, should have occurred in a period within 2,000,000 years, the K-Ar dating for the oldest subaerial lava flows known on the island [25]. It is unclear why other species of the genus *Orchestia *occurring in humid laurel forests on other Canary Islands (such as the endemic *O. guancha *from Tenerife and *O. gomeri *from La Gomera) have not colonized the subterranean habitat of their respective islands.

Another important issue concerns the dispersal potential of subterranean animals and whether apparently widespread subterranean species have evolved once and expanded their range by dispersal (with vicariance if there are no longer connections between extant populations) or evolve independently by convergent evolution from widespread surface populations in different parts of their range [26,27] The first case would lead to the display of convergent adaptations. This seems to be the case in the different *Astyanax *fish populations, the best studied case of adaptation to the subterranean environment in vertebrates [27]. In the facultative cave-dwelling crayfish *Cambarus tenebrosus*, the cave-dwelling and subterranean populations form a monophyletic group and clades are geographically but not habitat-structured, suggesting that there is (or has been) gene flow between the two habitats [6]. In the cave-dwelling landhopper *P. hypogaea*, Stock and Martin [22] claimed that the distribution of the species in lava tubes and caves all over La Palma and especially in tubes formed by recent lava flows (as recent as 300 years old as in Cueva del Ratón), would suggest that this talitrid can colonize new subterranean habitats quickly. Thus, a single colonization event followed by dispersal via the hypogean crevicular system could explain the observed pattern of habitats of these crustaceans.

We have used mitochondrial (mt) DNA and nuclear sequence data to establish a phylogenetic framework for testing hypotheses on the origin of *P. hypogaea *and to study the phylogeography of cave and surface populations of the genus *Palmorchestia *on the island of La Palma. In particular, we tested whether all populations of *P. hypogaea *could have derived from a common ancestral population that adapted to life in the volcanic tubes and dispersed through the volcanic subsoil, or whether there were multiple independent invasions of the caves from partially isolated, genetically differentiated populations of an ancestor presumably similar to the extant forest species *P. epigaea*.

## Results

**Figure 1:**
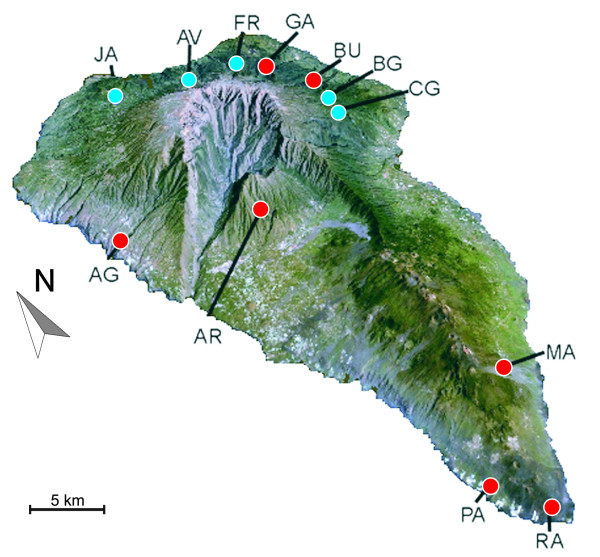
**Satellite photograph of the island of La Palma (obtained from NASA World Wind) with sampling sites for *P. epigaea *(blue) and *P. hypogaea *(red)**. Codes correspond to the localities indicated in Table 1.

A total of 89 *Palmorchestia *individuals were sequenced (53 *P. hypogaea *and 36 *P. epigaea*) for the subunits *cox1 *and *cox2 *of the cytochrome oxidase mtDNA region resulting in an alignment of 761 bp. The examined individuals of the two species showed many distinct haplotypes (43 for *P. hypogaea *and 25 for *P. epigaea*; accession numbers [EMBL: AM749302–AM749391]); see Table 1 and Table S1 in Additional file 1. Haplotypes were endemic to their respective cave or surface localities in the two species, with the exception of one haplotype of *P. epigaea *that was shared by the two nearby localities, Cubo de la Galga and Barranco de la Galga, at the north-east of the island (Figure 1). Therefore, no shared haplotypes between subterranean and surface species were evident in our samples. mtDNA haplotype diversity, measured by either species or locality, was high although sample sizes were low in some cases (Table 1). Only Barranco de la Galga and Andén Verde (*P. epigaea*) and Cueva de la Buraca (*P. hypogaea*) showed a fixed mtDNA genotype (the latter in the only two available individuals examined), but all other surface localities or lava tubes in which *Palmorchestia *was sampled rendered multiple haplotypes with a remarkably high rate of nucleotide variation.

**Table 1:** List of samples. List of examined species, sampling sites an their geographical positions, population codes (for La Palma localities of *P. epigaea *and *P. hypogaea*), numbers of individuals and the mtDNA haplotypes found. Haplotype diversities and their standard deviations are also shown. Tenerife, La Gomera, Gran Canaria, Fuerteventura and Lanzarote are islands of the Canarian archipelago.

**Taxa**	**Locality**	**Geographical position**	**Code**	**Number of individuals**	**Number of haplotypes**	**Haplotype diversity (± SD × 10^-1^)**
*P. hypogaea*	Cueva Honda de Gallegos	28° 49' 43" N 17° 43' 13" W	GA	8	8	9.81 ± 0.80
	Cueva de Palmeros de Aguatavara	28° 43' 59" N 17° 57' 57" W	AG	10	5	8.44 ± 0.80
	Cueva del Arenal	28° 40' 56" N 17° 51' 11" W	AR	6	4	8.00 ± 1.72
	Cueva de la Machacadora	28° 30' 30" N 17° 49' 42" W	MA	13	12	9.87 ± 0.35
	Cueva de los Palmeros	28° 30' 27" N 17° 51' 34" W	PA	8	7	9.29 ± 0.84
	Cueva del Ratón	28° 27' 46" N 17° 50' 47" W	RA	6	6	8.00 ± 1.72
	Cueva de la Buraca	28° 47' 29" N 17° 46' 43" W	BU	2	1	N/A
*P. epigaea*	Juan Adalid	28° 50' 21" N 17° 54' 27" W	JA	8	7	9.47 ± 2.00
	Andén Verde	28° 49' 51" N 17° 52' 07" W	AV	5	1	N/A
	Barranco de los Franceses	28° 48' 54" N 17° 51' 00" W	FR	6	6	9.52 ± 0.96
	Cubo de la Galga	28° 45' 47" N 17° 46' 35" W	CG	12	10	8.94 ± 0.78
	Barranco de la Galga	28° 46' 02" N 17° 46' 42" W	BG	5	1	N/A
*O. guancha*	Zapata (Tenerife)	28° 31' 56" N 16° 17' 27" W		10	8	7.56 ± 1.30
*O. gomeri*	Teselinde (La Gomera)	28° 11' 45" N 17° 17' 16" W		3	3	1.00 ± 2.72
*O. sp*	Andén Verde (Gran Canaria)	28° 02' 03" N 15° 45' 22" W		2	2	1.00 ± 5.00
*O. gammarellus*	Ajuí – Madre del Agua (Fuerteventura)	28° 24' 04" N 14° 08' 02" W		1	1	N/A
*O. stephenseni*	Famara (Lanzarote)	29° 12' 48" N 13° 29' 06" W		10	7	8.00 ± 1.72
*Talitroides alluaudi*	La Laguna (Tenerife)	28° 28' 31" N 16° 18' 36" W		3	1	NA

### Phylogenetic analyses

**Figure 2:**
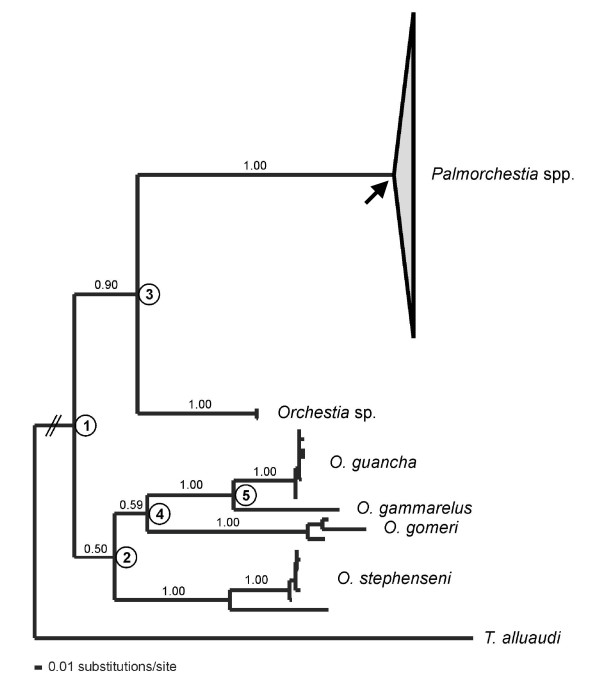
**mtDNA phylogenetic tree obtained by Bayesian inference analysis under a GTR + G + I model**. Base frequencies A = 0.3266, C = 0.2140, G = 0.0992, T = 0.3604; rate matrix A-C = 0.5706, A-G = 7.2807, A-T = 0.3662, C-G = 0.8075, C-T = 3.8068, G-T = 1.0000; gamma shape parameter G = 0.8533; proportion of invariant sites = 0.3085. Values above nodes correspond to posterior probability values greater than 0.85. The nodes numbered were used for age dating using a relaxed clock. Phylogenetic relationships among *Palmorchestia *populations are shown in detail in Figure 3. The arrow indicates the node that was used as calibration point for the estimations of divergence time.

**Figure 3:**
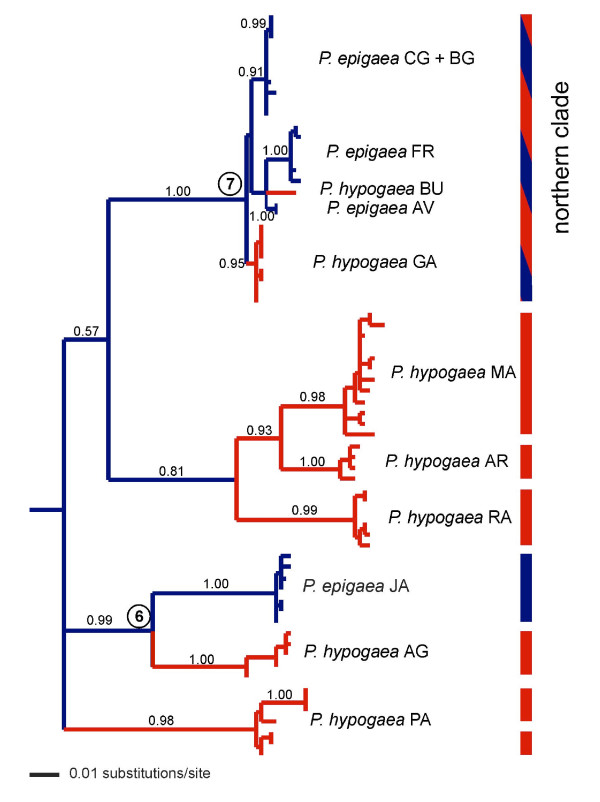
**mtDNA phylogenetic relationships obtained by Bayesian inference analysis for the *Palmorchestia *populations**. Values above nodes correspond to posterior probability values greater than 0.85. Branch colour coding in the tree shows parsimonious character optimization for habitat (blue, epigean; red, hypogean) assuming irreversibility. Bars to the right indicate the statistical parsimony mtDNA network membership, with blue bars corresponding to networks of pure *P. epigaea *haplotypes, red bars to networks of *P. hypogaea *haplotypes and the blue-red to the networks including haplotypes from both *P. epigaea *and *P. hypogaea *populations. The numbered nodes were used for age dating using a relaxed clock.

A sequence alignment was obtained for the *Palmorchestia *haplotypes plus the sequences obtained of 26 individuals from five related *Orchestia *species collected (see Table 1). One hundred and seventy-nine nucleotide positions were of the *cox1 *gene, up to 55 of an intergenic spacer, and 492 of *cox2*. The minimum in-group mean corrected distance among species (using the selected 'General Time Reversible with proportion of Invariant sites and a Gamma shape parameter' model, GTR + I + G) was 9.4% between *P. epigaea *and *P. hypogaea *and a maximum of 78% between *O. gomeri *and *P. hypogaea*. The sequence of the related landhopper *Talitroides alluaudi *was used as an out-group. Histone H3 sequences obtained from 20 individuals [EMBL: AM748646–AM748665] showed no variation within *Palmorchestia *or within the other *Orchestia *species sequenced while a 4.7% mean distance was obtained in comparisons between in-group species. mtDNA Bayesian inference and parsimony trees showed the same overall topology, which agreed with the among-species relationships deduced from nuclear histone H3 sequences (see Figures 2 and 3 for the mitochondrial Bayesian trees and Figure S1 in Additional file 1: for the combined tree). Each *Orchestia *species formed highly supported monophyletic mtDNA lineages, although relationships between species were weakly supported in most cases when using only mtDNA (Figure 2). A monophyletic clade composed of all *Palmorchestia *sequences appears as a sister group to *Orchestia *sp. from Gran Canaria (supported with a 0.90 posterior probability using mtDNA but 1.00 in the combined mtDNA + nuclear analysis). Interestingly, the sequences of *P. hypogaea *and *P. epigaea *did not form reciprocally monophyletic clades when using mtDNA (Figure 3), yet all examined individuals of these species showed identical histone H3 sequences. The clade formed by sequences of a *P. epigaea *population (JA) is sister to the *P. hypogaea *Palmeros de Aguatavara cave (AG) clade (0.99 posterior probability) and two *P. hypogaea *cave populations (GA and BU) cluster with the mtDNA haplotypes of the remaining four *P. epigaea *surface populations of the same geographical region (1.00 posterior probability; see Figure 3). All but two surface localities (CG and BG) showed support for monophyletic mtDNA clades (posterior probabilities over 0.95). This suggests that the *Palmorchestia *populations are highly structured geographically and have diverged genetically. Moreover, there is evidence that current gene flow among surface populations is limited to geographically close populations. Parsimonious reconstructions of habitat type based on the mtDNA analysis showed a minimum of five transitions to the subterranean habitat from epigean ancestors (Figure 3), assuming that reversal to the surface of hypogean lineages is absent or very unlikely. Alternatively, if transition between the two habitats was considered, four hypo-epigean shifts in the phylogeny could be deduced (not shown), but for this scenario to hold a subterranean lineage that became extinct or has not yet been found must be invoked.

### Divergence rates and molecular clock

Based on a relaxed molecular clock Bayesian analysis and an *a priori *calibration for the node of *Palmorchestia *using La Palma subaerial age, the mean substitution rate was estimated to be in the range of 4.34–4.83% per lineage per million years using a Yule and coalescent size models as priors, respectively (Table 2). This results in an average substitution rate of 4.6 × 10^-8 ^per site per year for the *cox1–cox2 *fragment used with 95% highest posterior densities (HPDs) in the range 2.9–7.2 × 10^-8^. Similar divergence time estimates for the major cladogenetic events were obtained irrespective of the model used except for the tree root node that was estimated to be significantly more recent using the Yule process than when coalescent models were applied (Table 2). The constant population size coalescent model had the best likelihood value (see Table 2). Within *Palmorchestia*, the most recent common ancestor for the subterranean lineage AG and the surface population JA was estimated at 940,000 years with 95% HPD values of 460,000–1,430,000 years assuming a constant population size model. The most recent common ancestor (MRCA) of the surface-subterranean northern clade including BU + GA + FR + AV + CG + BG haplotypes (node 7 of Figure 3) would be dated at an estimated mean of 360,000 years (95% HPD 170,000–580,000 years). The MRCA of cave populations spanned from a minimum of 70,000 years for GA (95% HPD 20,000–150,000) to a maximum of 310,000 years for PA (95% HPD 120,000–540,000) (Table 2). More recent coalescent times were estimated for the forest populations. This shows that the adaptation to cave life is relatively ancient for the age of La Palma.

**Table 2:** Estimation of coalescence times. Bayesian analysis and parameter estimation using a non-correlated relaxed molecular clock and dating of major clades in the trees of Figures 2 and 3 assuming a Yule tree prior and alternative coalescent population growth models. Mean and 95% HPD values are indicated in millions of years.

		**Coalescent model**	
		
**Clades**	**Yule model**	**Constant size**	**Exponential growth**
Tree root	9.86 (6.92–13.10)	14.19 (5.44–23.99)	14.61 (6.63–23.48)
Node 1	7.16 (5.30–9.24)	7.88 (4.31–11.79)	8.30 (5.2–12.09)
Node 2	4.96 (3.47–6.54)	5.30 (2.49–8.21)	5.84 (3.18–9.13)
Node 3	6.82 (4.65–9.10)	6.23 (3.22–10.10)	6.85 (3.79–10.71)
Node 4	4.31 (2.95–5.83)	4.17 (1.89–6.64)	4.77 (2.27–7.46)
Node 5	2.35 (1.56–3.18)	1.99 (0.77–3.40)	2.20 (0.88–3.54)
Node 6	1.04 (0.69–1.39)	0.94 (0.46–1.43)	0.89 (0.43–1.36)
Node 7	0.33 (0.21–0.47)	0.36 (0.17–0.58)	0.32 (0.16–0.51)
*P. epigaea *CG + BG	0.12 (0.06–0.19)	0.11 (0.04–0.20)	0.10 (0.04–0.17)
*P. epigaea *FR	0.09 (0.04–0.16)	0.09 (0.03–0.16)	0.08 (0.03–0.15)
*P. epigaea *AV	0.02 (0.00–0.06)	0.02 (0.00–0.06)	0.01 (0.00–0.04)
*P. hypogaea *GA	0.08 (0.02–0.14)	0.07 (0.02–0.15)	0.07 (0.02–0.13)
*P. hypogaea *MA	0.27 (0.17–0.38)	0.28 (0.13–0.46)	0.25 (0.12–0.40)
*P. hypogaea *AR	0.18 (0.09–0.29)	0.18 (0.06–0.34)	0.17 (0.05–0.31)
*P. epigaea *JA	0.11 (0.05–0.18)	0.11 (0.04–0.21)	0.10 (0.03–0.18)
*P. hypogaea *AG	0.22 (0.10–0.34)	0.23 (0.08–0.42)	0.21 (0.07–0.36)
*P. hypogaea *PA	0.29 (0.15–0.43)	0.31 (0.12–0.54)	0.28 (0.12–0.48)
Tree log-normal likelihood	-6193.1 (-6192.6; -6175.3)	-6163.9 (-6181.3; -6146.4)	-6165.4 (-6184.6; -6148.4)
Mean substitution rate (% per million years)	4.34 (3.18–5.54)	4.83 (2.72–7.22)	4.68 (2.91–6.73)

### Analysis of molecular variance

Analysis of molecular variance (AMOVA) showed that variance among groups maximized when partitioning *Palmorchestia *samples considering the north-east localities as a single population and the remaining sites as distinct populations (79.96%, see Table 3), in accordance with the tree topology. Partitioning the populations in subterranean and surface habitat groups produced a very low variance between groups (12.3%), whereas most variance was attributable to differences among populations within groups (78.22%). This demonstrates that there has been no significant association between genetic variation and habitat, but a strong association of haplotypes with island geographic realms irrespective of their surface or troglobitic life style.

**Table 3:** AMOVA analyses. Regional structure of *Palmorchestia *populations when localities are grouped based on geography or habitat (surface or cave). Fixation indices *Φ*_ST _(within populations), *Φ*_SC _(among populations within groups), *Φ*_CT _(among groups of populations in the species) and the significance levels of *Φ*_CT _are shown.

	***Φ*-statistics**	**Percentage of total**	***P*-value**
	
Geography [JA, AV, FR, GA, CG, BG, GA] [AG] [AR] [MA] [PA] [RA]			
Within populations	0.556	8.90	<0.01
Among populations within groups	0.799	11.14	<0.01
Among groups	0.799	79.96	<0.01
Habitat surface/subterranean [JA, AV, FR, CG, BG] [GA, BU, AG, AR, MA, PA, RA]			

Within populations	0.902	9.75	<0.01
Among populations within groups	0.889	78.22	<0.01
Among groups	0.120	12.03	Not significant

### Statistical parsimony

**Figure 4:**
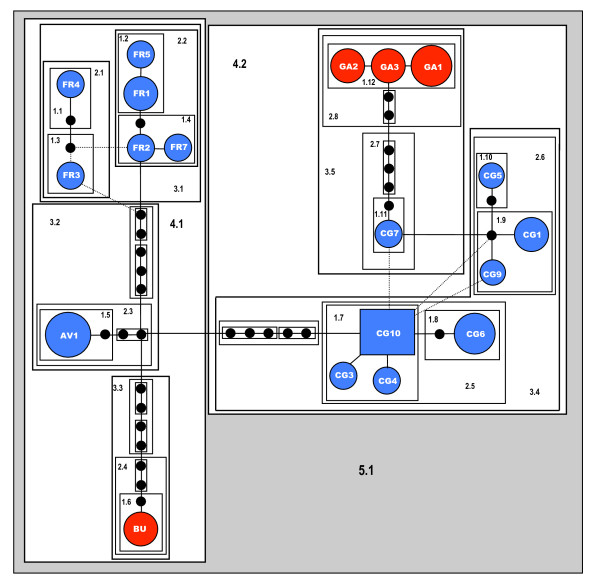
**Statistical parsimony network obtained with TCS for the composite surface/subterranean northern clade**. Each connecting line represents a single mutational step between any two given haplotypes. Dotted lines represent alternative ambiguous connections (loops). Black circles represent haplotypes not sampled (either extinct or not found in our sample), with the size of the circle of sampled haplotypes scaled approximately to the number of individuals possessing that haplotype. The nested design is represented by increasing levels of nested boxes, labelling the nesting level with the first number and particular clades at this level with the second one. Blue haplotypes correspond to *P. epigaea *(locality codes as in Table 1 and Figure 1) and red haplotypes correspond to *P. hypogaea*.

Network diagrams represent intraspecific evolution better than phylogenetic bifurcating trees [28]. Analysis of the *Palmorchestia *data set with the program TCS showed 12 as the maximum number of substitutions before homoplasy was likely to occur under the 95% probability criterion. This produced eight separated sub-networks above this limit, corresponding to the main clades of the tree of Figure 3. A cave locality (Cueva de los Palmeros, PA) has two disjoint sub-networks (Figure 3), suggesting that two distant mtDNA lineages occur within the same cave system. The rest of the cave localities and the epigean Juan Adalid (JA) north-west sampling site showed specific separate networks and thus highly distinct mtDNA lineages. Only one sub-network (corresponding to the north-east clade) included mtDNA haplotypes united below the limit of parsimony from individuals coming from six localities: four from surface populations of the north-north-east part of the island and the caves Cueva Honda de Gallegos and Cueva de la Buraca (GA and BU, respectively), located in the same geographical area (see Figure 1). As mentioned above, the general pattern is consistent with considerable geographic structuring of both *P. epigaea *and *P. hypogaea *populations and indicates a strong allopatric fragmentation. Nested clade phylogenetic analysis (NCPA) could only be applied to the northern sub-network, corresponding to evolutionary recent events deduced to have occurred less than 370,000 years ago on average (see above). The haplotype network and nesting design for this lineage is represented in Figure 4. Haplotype CG10 appears central in the network and is the most frequent, as it is present in six of the 39 sampled individuals of this clade. This haplotype is also fixed in the BG population, and is present in the neighbouring CG locality. Nesting resulted in 12 one-step clades containing sampled haplotypes that were further nested into higher-order groups until the total cladogram was obtained. The higher-order groups included surface haplotypes from FR and AV plus the only haplotype of the BU cave (clade 4.1) and haplotypes from surface CG, BG and cave GA sampling sites (clade 4.2). The nested contingency analysis of geographic associations and the interpretations of the statistically significant clades lead to a 'contiguous range expansion' within clade 4.1 and a 'past gradual range expansion followed by fragmentation' within clade 4.2 (Table 4), assuming that haplotype CG10 (and the higher-order clades embracing it) is ancestral to any other haplotype in the north-east sub-network.

**Table 4:** GeoDis analyses. Nested contingency results for clades with genetic and geographical variation within the northern network based on 10,000 permutations (CRE, contiguous range expansion; PGRE-F, past gradual range expansion followed by fragmentation).

**Clade**	**χ^2^**	**Probability**	**Inference chain**	**Inference**
3.5	9.00	0.1075	No significance	N/A
4.1	28.00	0.0000	1-19-2-11-12-NO	CRE
4.2	20.91	0.0002	1-2-11-12-13-21-NO	PGRE-F
5.1	39.00	0.0000	No significance	N/A

### Habitat associations

Habitat association of mtDNA haplotypes (surface and cave-dwelling) was confirmed by permutation chi-squared tests for clades 4.1 (*χ*^2 ^= 14.00;*P *= 0.0115) and 4.2 (*χ*^2 ^= 20.91;*P *= 0.0002). In addition, there was no significant association of cave and surface haplotypes with the position (either terminal or interior) of those in the network (*P *= 0.28). These data suggest that there is no evidence supporting a recent colonization of the subterranean habitat by forest populations or *vice versa *on the north shield of the island.

## Discussion

The phylogenetic and population analyses clearly show that on La Palma there is strong phylogeographic structuring for *Palmorchestia *populations and that subterranean populations have multiple independent origins from surface ancestors. Divergent mitochondrial lineages are restricted to precise cave systems or surface ravines and regions. Analysis for all but two populations sharing the same mtDNA haplotype supported monophyletic clades and a DNA sequence variation that denoted an absence of current gene flow between populations; the exceptions were the BG and CG north-east surface closely placed localities. AMOVA confirmed this and showed that when grouping *Palmorchestia *populations by habitat type, the genetic variation between groups proved very low. However, variation was maximized when considering localities (caves and surface ravines) as distinct populations, except for the north-east cave and surface sampling sites that contain a single population. This, added to the lack of reciprocal monophyly of *P. epigaea *and *P. hypogaea *mtDNA sequences, suggests that any division of the two species by habitat and ecology is unnatural.

Nuclear histone H3 gene and mitochondrial sequences showed the close relationship of *Palmorchestia *species with the *Orchestia *sp. of Gran Canaria. The two *Palmorchestia *species currently considered are clearly paraphyletic and genetically quite distant from the other Canary endemic landhoppers of the genus *Orchestia*. Within the *Palmorchestia *lineage three mitochondrial clades were obtained. The relationship of these mtDNA lineages with geography and geological history requires an understanding of the volcanic evolution of the island. The geological development of La Palma has been studied in detail [25]. Two defined edifices can be distinguished [29]; the northern shield began to emerge over the sea at about 1.7–2.0 MYA and has undergone several volcanic cycles and terrain collapses that ended about 0.2 MYA. The southern ridge of the island is the product of much more recent and intense volcanic activity, beginning 0.12 MYA and lasting until the present. Thus, the southern half of La Palma, presumably including the shallow subsoil, is dominated by recent lava flows, some of them of in historic times. Only one of the three major mtDNA lineages within *Palmorchestia *is exclusive of hypogean populations, including a central and relatively more ancient lava tube (AR) and two southern younger caves (RA and MA). The other two lineages include surface and cave populations, one lineage from the north (AV, FR, GA) and the other from the north-east localities (CG, BG and BU) on the older northern shield, but surprisingly showing lower genetic divergences than the other lineages. The third mtDNA lineage includes south-west (PA), north-west (JA) and west (AG) populations. This shows that intraspecific genetic variation is clearly not directly linked to subterranean or surface habitats and that its geographic distribution cannot be explained only by the geological history of the island. In a phylogeographic study of the La Palma weevil *Brachyderes rugatus*, initial predictions based on the geological history of the island also proved to be too simple to explain the phylogeographic history of the species [29].

Divergence time estimations gave a faster molecular rate than other calculations made for stygobiont crustaceans using cytochrome oxidase subunit 1 (i.e. 1.25 × 10^-8 ^substitutions per lineage per position per year [30]). Faster rates have nevertheless been suggested for subterranean Australian amphipods [7]. However, mutation rates could be slower in subterranean organisms owing to longer generation times [31]. Cave-dwelling organisms have lower metabolic, growth, fecundity and fertility rates and thus longer generation times than their surface relatives [31,32]. Stygobiont amphipods and isopods need 8–10 years to reach sexual maturity, whereas their surface relatives only required 1–2 years [32,33]. Accordingly, 8 years was assumed as the generation time for Australian stygobiont amphipods of the genus *Pilbarus *and *Chydaekata *[7]. Similarly, in decapod crustaceans, generation times of 2 and 10 years for surface and subterranean closely related species, respectively, have been used [3], based on data published elsewhere [34,35]. In addition, it has been debated whether there is a relationship between the rate of molecular evolution and sampling time [36-38]. However, several factors other than intrinsic problems with lineage rate variation and branch length estimation can produce overestimations of the molecular rate when dating nodes based on island ages [38]. These include uncertainty of K-Ar dating caused by the burial of earlier lava flows resulting in underestimation of island age, population genetic variation within the ancestral island population and lineage extinction [38]. We have obtained a reasonably good sampling of La Palma populations but there is a long branch in the phylogenetic tree joining the MRCA node of the sampled *Palmorchestia *populations and the sister species occurring in Gran Canaria, making it feasible that extinction could be a factor that has inflated our rate estimate. Nevertheless, mean time estimates for epi-hypogean transitions (or the contrary if we assume habitat reversion as a possibility) for two supported nodes in the Bayesian mitochondrial tree that relate surface and subterranean populations ranged from 0.36 to 0.94 MYA in the northern and western lineages, respectively. This suggests that independent episodes of colonization of the underground from surface pre-diverged lineages have probably occurred repeatedly in La Palma at different times during the Pleistocene and are probably related to documented drought episodes in the Canaries [23].

According to Stock's hypothesis [21], one can speculate on the role played by the relative drought of the island in the local extinction of surface *Palmorchestia *populations, except in permanently humid zones such as the scattered ravines radiating down the volcano (in particular, on the northern slopes exposed to north-east trade winds). In relation to this, two general competing hypotheses have been proposed to explain the transition from surface to subterranean life: the 'climatic relict hypothesis' (CRH) [24] and the 'adaptive shift hypothesis' (ASH) [39-41]. In the CRH, species pre-adapted to the cave environment (i.e. living in leaf litter or under stones) invade the hypogean habitat, with the epigean populations becoming extinct because of subsequent climatic change. In contrast, the ASH assumes active colonization of caves and parapatric speciation accompanied by adaptive differentiation and reduced gene flow between the epigean and hypogean populations. Support for both hypotheses has been obtained using mitochondrial phylogenies and molecular clock approaches (i.e. [9,42]), although the lack of likely surface ancestral lineages and robust phylogenies makes it difficult to test them in particular cases [4]. The pattern obtained in *Palmorchestia *suggests a recurrent entrance into the cave systems by already highly structured surface populations with subsequent independent adaptation to the hypogean environment and eventual interruption of gene flow between the two habitats. Local extinction of surface populations as the climate became drier [23] and multiple invasions of the underground make the pattern more consistent with the CRH than with a parapatric speciation via adaptive shift. The apparent contradiction between the age of the surface lava flows and some of the mtDNA lineages deduced by molecular clock approaches would support this. No surface populations have been found in the southern ridge of La Palma, but caves in this area are home to *Palmorchestia *ancestral haplotypes that could have derived from recent subterranean dispersion from north-central island populations. Indeed, some of the surface lava flows above these caves are recent but sampling shows ancient mtDNA haplotypes with estimated coalescent times of about 100,000 years, whereas another cave (Cueva de la Machacadora) harbours divergent lineages. If the lava tubes in these young island regions were coetaneous to surface lava flows, landhopper populations in these systems should be considered as recent newcomers and, at least in some cases, the product of independent colonizations. However, the sampling scheme has probably not covered all of the existing populations, including only a partial representation of the underground biodiversity, as access to this habitat is limited to the known entrances to the lava tubes accessible to humans (usually produced by roof collapse). In contrast, cavehoppers can easily disperse through cracks and crevices of the lava flows (the mesocaverns [31]), quickly colonizing the new cave systems underneath older terrains, either epigean or hypogean. This renders a scenario in which episodic colonizations involved dispersal from the surface to the underground, followed by recurrent range expansion and colonization through the subsoil, whereas on the surface isolation was caused by distance and fragmentation because of the patchy nature of the suitable habitat.

Eight independent networks were obtained using statistical parsimony (Figure 3). Five of these were site-specific and separated by many mutational steps, implying long periods of independent evolution. The north-east *Palmorchestia *mitochondrial lineage, which includes several surface populations and two lava tube populations, provides an opportunity to infer recent evolutionary events and relationships between lineages from the two habitats. This clade has an estimated age of 360,000 years using our relaxed molecular clock calibration (Figure 3 and Table 2), being the only clade that includes all mtDNA haplotypes from different populations into a single network under the limits of parsimony. Application of NCPA to this network showed an inference of contiguous range expansion from the northern surface (AV + FC) to north-east cave (BU) populations and previous gradual range expansion followed by fragmentation for the clade including populations BG + CG and the distant cave GA (Figure 4 and Table 4). Complex interactions either among populations within one habitat or among populations of the two habitats plus incomplete sampling and low sample size for some localities could partially explain why the significant associations found are few and dependent on the clade level (and thus the relative age of particular clades). In a similar study of the facultative cave-dwelling crayfish *Cambarus tenebrosus*, restricted gene flow and contiguous range expansion were the more frequent inferences, explaining the considerably and unusually large distribution of this species [6]. This kind of pattern could occur frequently in populations of facultative subterranean organisms that have remained isolated for a sufficient time, but are lost or not evident in populations with long histories of isolation, as occurs in many of the *Palmorchestia *populations. Tests of association of haplotypes with habitat type or positioning in interior or terminal nodes in the network show that haplotypes are habitat-specific, but *Palmorchestia *has been present in both caves and surface habitats for a long evolutionary time. This can be deduced from the fact that ancient clades often occur in caves and in the network within the northern clade by the lack of statistical support for an association of surface haplotypes with interior nodes and of subterranean haplotypes with terminal nodes.

An added complication posed by cave fauna is the absence of morphological differentiation among divergent genetic lineages, resulting in the presence of cryptic species. This is in part caused by a high level of convergent evolution linked to the adaptation to darkness and has been shown recently for stygobiont amphipods [4,7,8]. In *Palmorchestia*, the independent evolution of populations for a considerable evolutionary time and fast molecular evolution are apparently uncoupled with morphological divergence. These results indicate that a re-evaluation of the taxonomic status of the current species of *Palmorchestia *is needed, with a revision of the presumed diagnostic morphological characters that differentiate *P. epigaea *and *P. hypogaea *and a search for characters that could identify individuals from different surface geographical regions and/or cave systems. If, as genetic data suggest, multiple entries to the subsoil have occurred in *Palmorchestia*, convergent morphological adaptation by eye degeneration and body depigmentation could have arisen independently by different mutations, as has been shown to occur in *Astyanax fasciatus *[27].

## Conclusion

The phylogenetic and population analyses of *Palmorchestia *from La Palma show the need for a rigorous revision of the taxonomic status of the genus, its evolutionary relationship with other Talitridae, and the morphological characters in which the two currently considered species differ. The island populations show an ancient independent evolution and there is evidence for multiple invasions of the volcanic cave systems that have historically acted as refuges. The data obtained supplement recent genetic studies on subterranean amphipod fauna [4,7,8], with the potential of using these new systems to test classical hypotheses of modes of evolution for hypogean organisms. These include causes of the uncoupling of morphological and genetic evolution and the role of climatic change, hydrology and regressive evolution as processes shaping and determining subterranean biodiversity.

## Methods

### Sampling

Individuals of *P. epigaea *and *P. hypogaea *were collected in La Palma (Canary Islands) in 2005 and 2006. The troglobitic individuals (*P. hypogaea*) were collected in six lava tubes by direct active searches, or with pitfall traps using liver bait containing propylene glycol (Table 1). Individuals collected by active search were transferred immediately to absolute ethanol or RNAlater vials (Qiagen, Inc., Valencia, CA, USA). Traps were revisited after several weeks or months and individuals transferred to ethanol. The epigean species was formerly known to occur in a single locality (Cubo de La Galga), but after intensive searching four additional populations were discovered and included in this study (Table 1). Thus, epigean individuals (*P. epigaea*) were collected in five localities (two of them, Cubo de la Galga and Barranco de la Galga, are close to each other representing ravines separated by a crest) after searching under rocks or in litter in the dark humid forest between 500 and 1000 m above sea level. Sampling sites are shown in Figure 1. Details of the localities, numbers of individuals and numbers of haplotypes found appear in Table 1. The sampling regime was designed to cover different areas of the island (Figure 1), including most of the known distribution range of the species. The aim was to sample at least 10 specimens per locality, but this was possible only for two caves and one surface locality (or two if we consider a single population to constitute the samples from two close areas; Table 1). The related Canary landhoppers *O. guancha *(endemic to Tenerife), *O. gomeri *(endemic to La Gomera) and an undetermined *Orchestia *species from Gran Canaria were also sampled. Other sublittoral species of wider geographic distribution (*O. gammarellus, O. stephenseni *and the cosmopolitan *T. alluaudi*) were also considered for phylogenetic analyses (Table 1).

### DNA extraction, polymerase chain reaction amplification and sequencing

DNA was extracted using Qiagen DNAeasy Tissue kit (Qiagen); in most cases voucher specimens were preserved for morphological analysis. A mitochondrial fragment of about 800 bp including the 3' end of the *cox1 *sequence, the 5' of *cox2 *and the intergenic spacer between the two (as the tRNALeu nucleotide sequence (UUR) seems to be absent in the region in these species [43]) was amplified using primers SUBIF 5'AAGAGGCATACCTCGACGATACTC3' and COII-CROZ 5'CCACAAATTTCTGAACATTGACC3' primers [43,44]. Polymerase chain reaction (PCR) conditions were as follows: 4 min at 95°C followed by 35 cycles of denaturation at 95°C for 30 s, annealing at 50°C for 1 min and extension at 72°C for 2 min, with a final single extra extension step at 72°C for 10 min. A fragment of the nuclear histone H3 gene (using the primers forward 5'ATGGCTCGTACCAAGCAGACVGC3' and reverse 5'ATATCCTTRGGCATRATRGTGAC3' [45]) was also amplified successfully in several individuals of *Palmorchestia *from different cave and surface localities and in the out-group taxa. PCR conditions were the same as that for the mitochondrial fragment. PCR products were checked by electrophoresis in 1% agarose gels and products of the expected lengths were purified using the QIAquick PCR Purification Kit (Qiagen). The forward and reverse strands were cycle-sequenced using an ABI Prism DYE Terminator Cycle Sequencing Reaction Kit sequenced in an ABI 3100 automated sequencer (Applied Biosystems, Foster City, CA, USA).

### Phylogenetic analyses

Sequences were aligned with ClustalX [46]; length differences in the alignments comparing different species were found to be caused by indels in the intergenic spacer. Bayesian inference analysis was performed with MrBayes 3.1.1 [47] using the substitution model(s) obtained by ModelTest v. 3.7 [48]. The analysis for the entire combined dataset was conducted with particular optimal models fitted for each of the mitochondrial (GTR + I + G) and histone H3 (HKY + G) partitions. The parameters corresponding to the selected model were treated as unknown variables with equal *a priori *probability and estimated as part of the analysis based on Bayesian inference. Searches were performed using two runs of 2,000,000 generations from four Markov independent chains started from random seeds sampling every 500 generations. At the end of each run we considered the sampling of the posterior distribution to be adequate if the average standard deviation of split frequencies was less than 0.01. Markov chain Monte Carlo (MCMC) runs were summarized and further assessed for convergence of all parameters, using the program Tracer v.1.3 [49]. Trees prior to log likelihood stabilization (burn in) and convergence were discarded before producing a majority rule consensus tree. Parsimonious reconstructions of habitat type in the mtDNA topology were performed using MacClade v. 3.03 [50] assuming reversibility or irreversibility of character states, thus constraining or not constraining subterranean clades to reverse to an epigean habitat.

### Molecular rate and divergence times

We used a likelihood ratio test to determine whether a molecular clock in the whole mtDNA sequence data set would be compatible with the hypothesis of a global clock. The null hypothesis was rejected at *P *= 0.01. Thus, a full Bayesian analysis was performed to estimate evolutionary rates and coalescent times of *Palmorchestia *lineages, as implemented by the program Beast v. 1.4.6 [51]. The molecular clock assumption was relaxed using uncorrelated rates, with the rate in each branch independently drawn from a log-normal distribution with a mean of 0.01 and a variance parameter of 0.5. For the analyses two coalescent population models were tested (constant size and exponential growth) plus the Yule tree prior. Final rate estimates from the three alternatives were compared and that showing the best likelihood score was chosen. A normally distributed calibration prior was set for the age of the *Palmorchestia *clade based on the La Palma subaerial formation with a mean of 2,000,000 years [25] and standard deviation of 200,000, thus assuming uncertainty associated with the calibration point. The mtDNA alignment was analysed using the best-fit substitution model obtained with ModelTest (GTR + I + G), 5,000,000 MCMC, sampled every 500 steps, following a discarded burn-in of 500,000 steps. For the parameters substitution model, we placed uniform priors of 0 and 100 except for the proportion of invariant sites in which uniform priors of 0 and 1 were assumed. Convergence of the chains to the stationary distribution was assessed by visual inspection of plotted posterior estimates using the program Tracer v. 1.3 [49] and the effective sample size for each parameter sampled from the MCMC analysis was almost always found to exceed 100.

### Population analyses

AMOVA using Arlequin v. 2000 [52] was performed on the *Palmorchestia *mtDNA data set to partition molecular variance into different hierarchical levels. A matrix of pairwise Euclidean squared distances between haplotypes and files containing the frequency of those haplotypes within each population were used for this purpose. We tested for differences between sampling sites nested within regional groups and differences between groups. The significance of the variance components was evaluated by a non-parametric permutation test [53] using 10,000 permutations. Populations were grouped by habitat (subterranean versus epigean) or by sampling localities: the latter irrespective of species. The grouping that maximized the geographical subdivision was assumed as the most plausible within those supported by significant variation among regions (*Φ*_CT_).

TCS v. 1.13 [54] was used to infer the parsimony network of haplotypes, linked by the smallest number of mutational steps with a 95% confidence criterion [55]. NCPA [56] could not be performed in most cases because the haplotypes contained in the networks were largely allopatric and disjunctive networks were far beyond the limit of parsimony at the 95% level. Haplotype nesting into hierarchical clades and NCPA was only performed in a sub-network including haplotypes of individuals from two subterranean and four surface localities. As suggested in [6], habitat association of mtDNA haplotypes (surface or subterranean) and significant relationships of subterranean or surface haplotypes with their peripheral or interior placements in this sub-network were tested by permutation chi-squared tests performed with GeoDis v. 2.4 [57]. Such analysis can reveal whether the surface populations invaded the subterranean environment recently and whether the surface haplotypes are more frequently interior and subterranean forms appear as tip and recent. Moreover, it can show whether surface populations are recent derivatives of cave ancestors. Finally, an ancient settlement (at the time scale examined) of both subterranean and surface populations was favoured if no significant association was found [6].

## Authors' contributions

CV carried out the molecular genetic studies, participated in phylogenetic analyses and collected most of the samples. DJ participated in the design of the study, helped with sampling and performed taxonomic work. PO and CJ conceived of the study, participated in its design and coordination and helped in sampling. CJ also participated in phylogenetic analyses and wrote the first draft of the manuscript. All authors read and approved the final manuscript.

## Supplementary Material

Additional file 1 **Supplementary table and figure**. **Table S1**: List of EMBL accession numbers for the haplotypes of *Palmorchestia *populations and the other species examined. **Figure S1**: Bayesian tree obtained in a combined analysis using mtDNA and nuclear histone H3 sequences. Values above nodes correspond to posterior probability values. Click here for file
