# Supplementary material for: Under the volcano: phylogeography and evolution of the cave-dwelling Palmorchestia hypogaea (Amphipoda, Crustacea) at La Palma (Canary Islands)
Source: BMC Biol. 2008 Jan 31;6:7. doi: 10.1186/1741-7007-6-7 (PMC2246103; doi:10.1186/1741-7007-6-7)

Table S1. List of EMBL accession numbers for the haplotypes of *Palmorchestia* populations and other species examined.

**Taxa**	**Population code**	**Acc. numbers Mitochondrial**	**Acc. numbers Histone H3**
Palmorchestia	GA	AM749316-21	AM748651
hypogaea		AM749329-30	
	AG	AM749379-83	AM748653
	AR	AM749305-08	AM748649-50
	MA	AM749302-04	--
		AM749311-18	
	PA	AM749372-78	--
	RA	AM749322-28	AM748652
		AM749331	
	BU	AM749391	--
			--
P. epigaea	JA	AM749384-90	--
	AV	AM749370	--
	FR	AM749364-69	AM748647
	CG	AM749332-41	AM748648
	BG	AM749340	--
Orchestia guancha		AM749342-49	AM748654-57
O. gomeri		AM749353-55	AM748658-59
O. sp		AM749358-59	AM748660-61
O. gammarellus		AM749356	AM748665
		AM749350-51	AM748662-64
O. stephenseni		AM749357	
		AM749360-63	
Talitroides alluaudi		AM749352	AM748646

Figure S1. Bayesian tree obtained in a combined analysis using mitochondrial DNA and nuclear histone H3 sequences. Values above nodes correspond to posterior probability values.

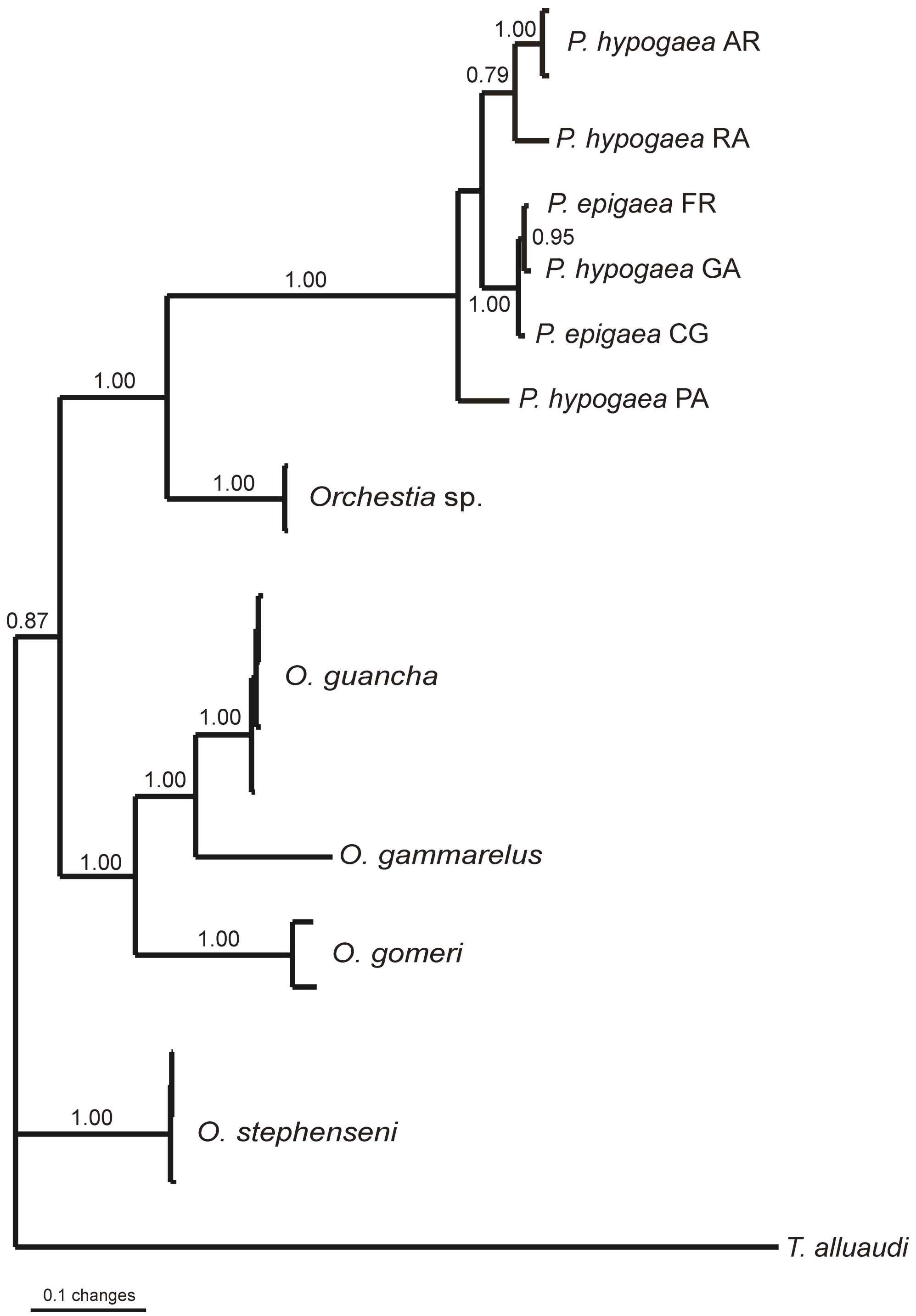

Supplement: Additional file 1 — Supplementary table and figure. Table S1: List of EMBL accession numbers for the haplotypes of Palmorchestia populations and the other species examined. Figure S1: Bayesian tree obtained in a combined analysis using mtDNA and nuclear histone H3 sequences. Values above nodes correspond to posterior probability values. [file 1741-7007-6-7-S1.PDF]
